# Etiology of isolated pontine infarctions: a study based on high-resolution MRI and brain small vessel disease scores

**DOI:** 10.1186/s12883-017-0999-7

**Published:** 2017-12-12

**Authors:** Cheng Xia, Hui-Sheng Chen, Shi-Wen Wu, Wei-Hai Xu

**Affiliations:** 1 Department of Neurology, General Hospital of Shenyang Military Command, 83 Wen Hua Road, 110840 Shenyang, People’s Republic of China; 2 Department of Neurology, General Hospital of Chinese Armed Police Forces, Beijing, China; 3 Department of Neurology, Peking Union Medical College Hospital, Chinese Academy of Medical Sciences, Beijing, China

**Keywords:** Pontine infarction, Pathogenesis, HR-MRI, Small vessel disease score

## Abstract

**Background:**

In this retrospective study, we investigated the main pathogenesis of the two types of isolated pontine infarction: paramedian pontine infarcts (PPIs) and small deep pontine infarcts (SDPIs).

**Methods:**

Acute ischemic stroke patients, comprising 117 PPI patients and 40 SDPI patients, were enrolled. High-resolution magnetic resonance imaging (HR-MRI) and routine MRI sequences were performed for each patient, and clinical data were collected. The following brain small vessel disease (SVD) features of the MRI scans were each rated (0 or 1) separately: asymptomatic lacunar infarcts, white matter lesions (WMLs), deep and infratentorial cerebral microbleeds (CMBs), and enlarged perivascular spaces in the basal ganglia. The ratings were also summed in an ordinal “SVD score” (range: 0–4). The difference in the SVD score between the PPI and SDPI groups was determined. The presence and location of basilar artery (BA) atherosclerotic plaques (based on HR-MRI) in the two groups was evaluated.

**Results:**

There was a significant difference in the total SVD score and three of the four independent SVD features (asymptomatic lacunar infarcts, WMLs, and deep and infratentorial CMBs) between the two groups. The prevalence of BA plaques relevant to the infarcts in the PPI group was significantly higher than that in the SDPI group, whereas the prevalence of plaques irrelevant to the infarcts was similar between the two groups. The degree of BA stenosis was slightly higher in the PPI group than in the SDPI group. Diabetes mellitus was much more prevalent in the PPI group. The National Institute of Health Stroke Scale score was higher in the PPI group, which is in accordance with the larger infarct size in the PPI group.

**Conclusion:**

BA atherosclerosis may be the major cause of PPI, while SVD may be the main mechanism underlying SDPI. HR-MRI combined with the total SVD score should be helpful to explore the pathogenesis underlying isolated pontine infarctions, especially in cases involving low-grade BA stenosis.

## Background

Isolated pontine infarctions are usually classified into two types: paramedian pontine infarcts (PPIs) and lacunar pontine infarcts (LPIs), i.e., small deep pontine infarcts (SDPIs), according to the lesion shapes and locations [1, 2]. In PPI, the infarct abuts on the basal surface of the pons. In SDPI, the infarct does not reach to the surface of the pons [2].

So far, asymptomatic lacunar infarcts, white matter lesions (WMLs), deep and infratentorial cerebral microbleeds (CMBs), and enlarged perivascular spaces (EPVS) in the basal ganglia have all been identified as silent magnetic resonance imaging (MRI) markers of arteriolosclerotic cerebral small vessel disease (SVD), a pathological process involving the small arteries and arterioles of the brain [3–8]. Recently, a total SVD scoring system was developed, which involves summing the scores (0 or 1) for each of the various MRI features. It has been proved that this scoring system provides a simple and pragmatic overall score (range: 0–4), providing a more complete view of the impact of SVD on the brain than the individual MRI features [9–11].

In recent years, high-resolution MRI (HR-MRI) has been gradually used in more clinical studies. The advantage of HR-MRI is its high spatial resolution in displaying vessel walls, thus improving the power of MRI for helping to understand the pathogenesis of cerebral vascular disease. HR-MRI can be helpful to diagnose intracranial atherosclerosis [12], artery dissection [13, 14], Moyamoya disease [15], vasculitis [16], reversible cerebral vasoconstriction syndrome [17, 18], and radiation-induced intracranial vasculopathy [19].

The main pathogenesis of each of the two types of isolated pontine infarction has been investigated by several authors. Fisher and Caplan carried out individual case studies and suggested that the pathogenic mechanism of SDPI involved perforating small arterial disease caused by lipohyalinosis, whereas PPI was caused by atheromatous branch occlusion of the paramedian or circumferential basilar branch [20–22]. However, other in vivo studies concluded that SVD and BA plaques were both significant causes of PPIs [2, 23]. Possible reasons for the different conclusions include the limited sample sizes and defects in methodology. Thus, the underlying mechanisms warrant further investigations involving larger sample sizes and newly developed methods.

Based on the abovementioned issues, this study was designed to investigate the main causes of isolated pontine infarctions based on the total “SVD score” combined with HR-MRI findings.

## Methods

### Patient selection

We retrospectively reviewed 520 consecutively hospitalized patients with acute pontine infarction who were treated at our stoke center from October 2012 to August 2016. The inclusion criteria were as follows: ① First-ever symptomatic stroke; ② Acute isolated pontine infarction identified by diffusion-weighted imaging (DWI); ③ HR-MRI, magnetic resonance angiography (MRA), DWI, and susceptibility-weighted imaging (SWI) examinations completed within 7 days from onset. The exclusion criteria were as follows: ① Bilateral pontine infarction and/or infarction not confined to pons; ② Cerebral hemorrhage, subarachnoid hemorrhage, intracranial tumor, etc. ③ Infarction possibly caused by cardiac embolism, arteritis, artery dissection, vertebrobasilar dolichoectasia, etc. This study was approved by the Institutional Review Board of our hospital, however, informed consent was not required because it was a retrospective study.

### Clinical assessment

Clinical data were obtained by reviewing the patients’ electronic medical records. Demographics and stroke risk factors including age, sex, hypertension, diabetes mellitus, current drinking, current smoking, and history of cardiac disease (including coronary heart disease) were reviewed. All patients underwent a 12-lead electrocardiogram, and in selected cases, a specific cardiac workup, including 24-h Holter monitoring, transthoracic echocardiography, or transesophageal echocardiography, was performed to exclude the possibility of cardiac embolism. Laboratory test data included blood urea nitrogen (BUN), creatinine (Cr), glomerular filtration rate (GFR), Cystatin C (Cys-C), total protein (TP), albumin (ALB), serum total cholesterol (TC), triglyceride (TG), low-density lipoprotein cholesterol (LDL-C), high-density lipoprotein cholesterol (HDL-C), lipoprotein a (LP(a)), homocysteine (Hcy), fibrous protein (FIB), D-dimer, and high-sensitivity C-reactive protein (hsCRP). Stroke severity was measured using the National Institute of Health Stroke Scale (NIHSS) score at admission.

### Magnetic resonance (MR) protocols

The subjects were imaged with a 3.0-T MR scanner (GE Discovery MR 750 3.0-T, USA). The whole brain was scanned with a slice thickness of 6 mm in the axial and sagittal planes. The protocol consisted of T1-weighted images (repetition time [TR]/echo time [TE] = 1625/24), T2-weighted images (TR/TE = 4160/88), fluid attenuation inversion recovery (FLAIR; TR/TE = 8000/165), and DWI (TR/TE = 4000/68). 3D time-of-flight MRA scans (TR/TE = 19/2.3, slice thickness = 0.6 mm) were obtained in the axial plane. SWI scans (TR/TE = 36.8/24.3, slice thickness = 2 mm) were also obtained in the axial plane. Furthermore, black-blood HR-MRI sequence scans involving a T2 dark fluid spectral adiabatic inversion recovery (SPAIR) sequence (TR/TE = 3400/56, slice thickness = 1.5 mm) were obtained in the axial plane. HR-MRI T1 3D CUBE sequence scans (TR/TE = 575/15, slice thickness = 0.8 mm) were obtained in the coronal plane for 3D reconstruction.

### Imaging analysis

All images were reviewed by two experienced raters who were blinded to each patient’s clinical details. Any differences between the two raters were solved by consensus. According to the DWI, the type of isolated pontine infarction was classified as PPI or SDPI. In PPI, the infarct abuts the basal surface of the pons. In SDPI, the infarct does not reach the surface of the pons. The maximum diameter of the infarcts was measured by DWI using sagittal T2-weighted images. EPVS in the basal ganglia were evaluated using axial T2-weighted images [11]. WMLs were evaluated using FLAIR [24]. Asymptomatic lacunar infarcts were counted using axial T2-weighted images and FLAIR [9]. Deep (basal ganglia, thalamus, corpus callosum, internal capsular, external capsular, and periventricular white matter) and infratentorial (brain stem and cerebellum) CBMs were counted using SWI [25]. The total SVD score was obtained according to the ratings (0 or 1) regarding asymptomatic lacunar infarcts, WMLs, deep and infratentorial CBMs, and EPVS in the basal ganglia [9].

Basilar artery (BA) stenosis was measured by MRA. The degree of stenosis (%) = [(1 - (D stenosis/D normal))] × 100, where D stenosis = diameter of the artery at the site of the most severe stenosis, and D normal = diameter of the proximal normal artery. Asymptomatic pontine lacunes were evaluated using DWI and axial T2-weighted images. BA plaques were evaluated using HR-MRI T1 and T2 images. A plaque was identified if there was eccentric wall thickening; the thinnest part was estimated to be 50% of the thickest point by visual inspection [12]. BA plaques relevant to the infarcts were defined as those located in the proximal segment of the vessel relative to the infarcts, or those located on the dorsal wall or on the same side as the infarcts at the same level. The other BA plaques were defined as plaques that were irrelevant to the infarcts.

### Statistical analysis

SPSS 20.0 software (IBM Corp., Armonk, NY, USA) was used for the statistical analysis. Regarding the measurement data, if the data were normally distributed, they are presented as mean ± SD. If the data were not normally distributed, they are presented as median and interquartile range. For comparing continuous variables, t-tests or Wilcoxon rank sum tests were used. Count data is presented as n (%), and compared using Chi-square tests. The level of statistical significance was set at *P* < 0.05.

## Results

Of the 157 enrolled patients, there were 117 with PPI and 40 with SDPI. All the patients underwent MRI, MRA, DWI, and SWI scans, which amounted to 122 patients receiving HR-MRI scans. Regarding the observations of BA plaques, three patients were excluded due to poor-quality HR-MRI T1 and T2 images, so eventually 119 patients were retrospectively analyzed for BA plaques.The clinical characteristics are summarized in Table 1. The prevalence of diabetes mellitus in the PPI group (43.6%) was much higher than that in the SDPI group (17.5%). The median NIHSS score in the PPI group was also higher than that in the SDPI group. The laboratory test data between the two groups were similar, as summarized in Table 2.

**Table 1:** Clinical data of the study population

	All patients (*n* = 157)	PPI (*n* = 117)	SDPI (*n* = 40)
Age, y	62.1 ± 10.5	62.4 ± 10.3	61.2 ± 11.2
Male sex	115 (73.2)	83 (70.9)	32 (80.0)
Smoking	69 (43.9)	53 (45.3)	24 (60.0)
Drinking	73 (46.5)	55 (47.0)	18 (45.0)
DM	58 (36.9)	51 (43.6)	7 (17.5)^a^
Hypertension	115 (73.2)	83 (70.9)	32 (80.0)
IHD	23 (14.6)	17 (14.5)	6 (15.0)
NIHSS	3 (0-18)	4 (0-18)	2 (0-6)^a^

*IHD* ischemic heart disease, *DM* diabetes mellitus, *NIHSS* the National Institutes of Health Stroke Scale

Data presented as number (%), median (range) or mean ± SD

^a^ *p* < 0.05 PPI vs SDPI

**Table 2:** Laboratory data of the study population

laboratory data	PPI (*n* = 117)	SDPI (*n* = 40)	*P*-value
BUN (mmol/L)	5.48 (2.00)	5.60 (3.00)	0.915
Cr (umol/L)	65.00 (21.00)	68.35 (23.00)	0.372
Cys-C (mg/L)	0.88 (0.28)	0.88 (0.31)	0.493
GFR (ml/min)	109.69 ± 25.63	103.88 ± 27.69	0.231
TP (g/L)	69.20 ± 5.60	68.35 ± 5.17	0.402
ALB (g/L)	41.11 ± 3.60	40.50 ± 3.60	0.355
TG (mmol/L)	1.58 (1.00)	1.48 (1.00)	0.215
TC (mmol/L)	4.86 ± 1.17	4.58 ± 1.31	0.219
LDL-C (mmol/L)	2.89 ± 0.75	2.68 ± 0.82	0.134
HDL-C (mmol/L)	0.97 (0.00)	1.05 (0.00)	0.171
LP(a) (mg/L)	189.10 (181.63)	167.65 (196.00)	0.790
Hcy (umol/L)	12.73 (6.00)	13.59 (11.00)	0.499
FIB (g/L)	3.21 (0.84)	2.99 (1.04)	0.085
D-dimer (μg/ml)	0.30 (0.32)	0.32 (0.46)	0.814
hCRP (mg/L)	1.90 (2.61)	1.99 (1.65)	0.701

### MRI markers

The prevalence of asymptomatic lacunes in the SDPI group (55.0%) was higher than that in the PPI group (29.1%). The frequency of high-degree WMLs in the SDPI group (30%) was higher than in the PPI group (10.3%). The prevalence of deep and infratentorial CMBs was higher in the SDPI group (65.0%) than in the PPI group (36.8%). EPVS in the basal ganglia was similar in the two groups. The frequency of asymptomatic pontine lacunes in the SDPI group (45%) was higher than that in the PPI group (22.2%). As to the maximum infarct diameter, that in the PPI group (16.5 ± 4.7 mm) was larger than that in the SDPI group (8.1 ± 2.7 mm). The difference in the degree of BA stenosis between the PPI group (23%) and the SDPI group (13%) reached borderline significance (*p =* 0.064). All these data are summarized in Table 3.

**Table 3:** MRI markers for all patients and by stroke subtype

	All patients (*n* = 157)	PPI (*n* = 117)	SDPI (*n* = 40)
Lacunes	56 (35.7)	34 (29.1%)	22 (55.0%)^a^
WMLs	24 (15.3)	12 (10.3%)	12 (30.0%)^a^
CMBs	69 (43.9)	43 (36.8%)	26 (65.0%)^a^
EPVS	60 (38.2)	41 (35.0%)	19 (47.5%)
Pontine lacunes	44 (28.0)	26(22.2%)	18 (45.0%)^a^
Infarct diameter	14.4 ± 5.6	16.5 ± 4.7	8.1 ± 2.7^a^
BA stenosis	0.21 (0.26)	0.23 (0.27)	0.13 (0.30)^b^

*WMLs* white matter lesions, *CMBs* cerebral microbleeds, *EPVS* enlarged perivascular spaces, *BA* basilar artery

Data presented as number (%), median (interquartile) or mean ± SD

^a^ *p* < 0.05

^b^ *p =* 0.064

### Total SVD score

**Fig. 1:**
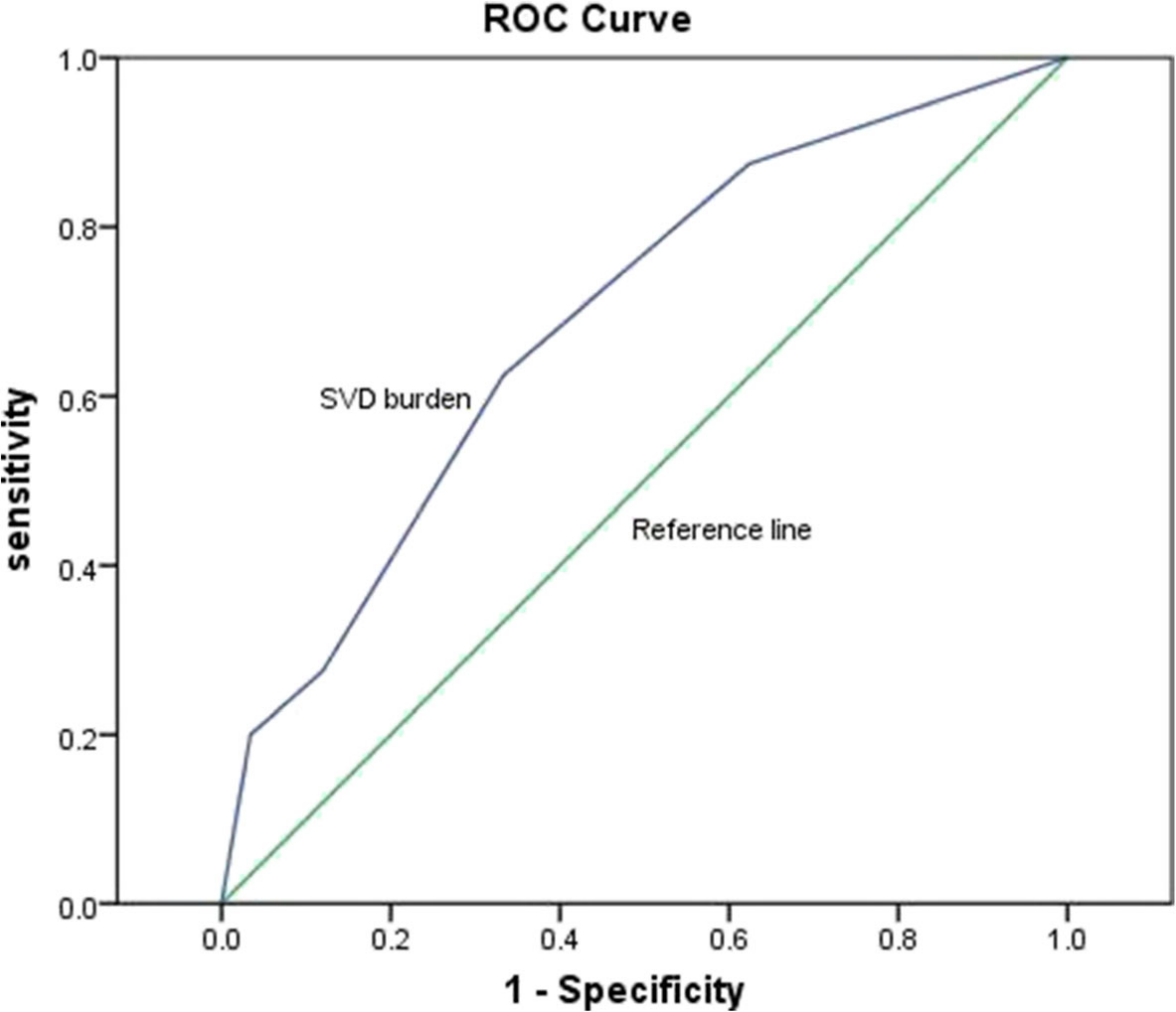
showing Roc curve of SVD score in predicting SDPI. The Area Under the Curve is 0.69 and the best cut-off value is 2 with a sensitivity of 0.625 and specificity of 0.667

Among the patients who scored 1, 34.1% had lacunes, 34.1% had CMBs, 31.8% had EPVS, and none had WHLs. Among those who scored 2, CMBs + EPVS (45.9%) was predominant, followed by lacunes + CMBs (32.5%), lacunes + EPVS (18.9%), and lacunes + WML (2.7%), but there was no EPVS + WML. Among those who scored 3, all potential combinations were present, and the frequencies were similar: EPVS + CMBs + lacunes (23.1%), WML + EPVS + CMBs (23.1%), WML + CMBs + lacunes (25.6%), and WML + EPVS + lacunes (28.2%). The SDPI group had a higher mean total SVD score than the PPI group (Table 4, *p* < 0.001). In predicting SDPI, the area under the curve (AUC) for the total SVD score was 0.69 and the best cut-off value was 2, with a sensitivity of 0.625 and a specificity of 0.667 (Fig. 1).

**Table 4:** SVD score values for all patients and by stroke subtype

SVD score	All patients (*n* = 157)	PPI (*n* = 117)	SDPI (*n* = 40)
0	49 (31.2)	44 (37.6)	5 (12.5)
1	44 (28.1)	34 (29.1)	10 (25.0)
2	39 (24.8)	25 (21.4)	14 (35.0)
3	13 (8.3)	10 (8.5)	3 (7.5)
4	12 (7.6)	4 (3.4)	8 (20.0)

*SVD* small vessel disease

Data presented as number (%). Mann-Whitney test PPI vs SDPI, *p* < 0.001

### BA plaques

**Fig. 2:**
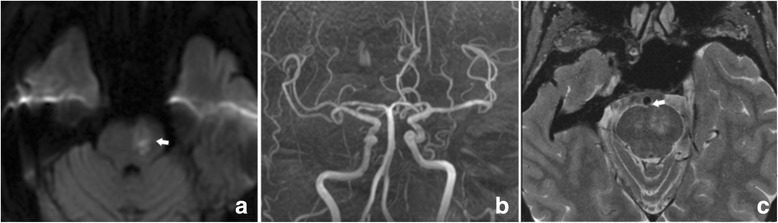
In a patient (60-69 year old) with acute isolated pontine infarction, (**a**) DWI showed paramedian infarct in the left pons (arrow); (**b**) MRA showed no obvious stenosis in the basilar artery; (**c**) T2 dark fluid SPAIR showed an atheromatous leison in the orifice of a paramedian artery (arrow)

**Fig. 3:**
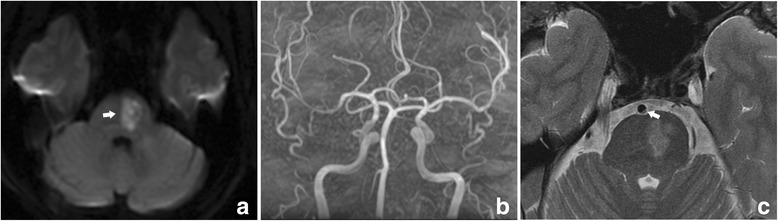
In a patient (50-59 year old) with acute isolated pontine infarction, (**a**) DWI showed a paramedian infarct in the left pons (arrow); (**b**) MRA showed no obvious stenosis in the basilar artery; (**c**) T2 dark fluid SPAIR showed a plaque relevant to the infarct (arrow)

**Fig. 4:**
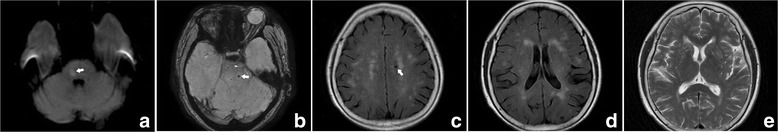
In a patient (70-79 year old) with acute isolated pontine infarction, (**a**) DWI showed a small deep infarct located in the right pons (arrow); (**b**) SWI showed one microbleed in the left pons (arrow); (**c**) FLAIR showed an asymptomatic lacune in the left centrum ovale (arrow); (**d**) FLAIR showed periventricular WMLs Fazekas 2; (**e**) T2 showed moderate perivascular spaces in basal ganglia. The total SVD score was 4

The prevalence of BA plaques irrelevant to the infarcts was similar in the two groups. The prevalence of plaques relevant to the infarcts was much higher in the PPI group (74.4%) than in the SDPI group (31.0%) (Table 5, *p* < 0.001). Examples of HR-MRI and SVD score images are illustrated in Figs. 2, 3 and 4.

**Table 5:** BA plaques for all patients and by stroke subtype

	All patients (*n* = 119)	PPI (*n* = 90)	SDPI (*n* = 29)
Relevant	76 (63.9)	67 (74.4)	9 (31.0)^a^
Irrelevant	52 (43.7)	40 (44.4)	12 (41.4)

Data presented as number (%). Pearson Chi-square test PPI vs SDPI. ^a^ *p* < 0.001

## Discussion

The results showed that BA atherosclerosis may be the major cause of PPI, while SVD may be the main mechanism underlying SDPI. To the best of our knowledge, this study is the largest retrospective study of an Asian population to explore the main mechanisms of the two types of isolated pontine infarction. For the first time, the total SVD score combined with HR-MRI was used in the investigation. There is no doubt that this will be helpful to understand the etiology of isolated pontine infarctions.

Previous studies have investigated the main causes of PPI and SDPI [1, 2, 20–23], but their conclusions were conflicting. We argue that there are two main limitations associated with these previous studies. Firstly, the sample size in some of them was small, so it is difficult to make an exact judgement about the major etiology of PPI and SDPI; secondly, the various studies also had methodological limitations. Although pathology is the gold standard for judging etiology, isolated pontine infarctions are rarely fatal, so it is difficult to explore the major causes from individual pathologic case studies. In one of the previous studies [23], although HR-MRI was used to detect BA plaques, only WMLs and lacunae were used as imaging markers of cerebral small vessel disease. Thus, in the present study, the total SVD score combined with HR-MRI was used to investigate the etiology of isolated pontine infarctions in a larger sample.

The results showed that the mean total SVD score was significantly higher in the SDPI group than in the PPI group, indicating that SVD is the main pathogenesis of SDPI. The prevalence of BA plaques relevant to the infarcts was much higher in the PPI group than in the SDPI group, while the frequency of irrelevant BA plaques was similar between the two groups, which indicates that BA atherosclerosis is significantly associated with PPI. These results imply that the main cause of PPI is probably large artery atherosclerosis, while SDPI may be attributable to SVD, which is in accordance with the findings of previous pathology-related research [21].

BA plaques were detected in 87.8% of the patients with PPI, which is higher than that in a previous report [26]. This might be due to application of the 3.0-T MR scanner (which is more sensitive for detecting plaques than a 1.5-T MR scanner), and also the high prevalence of intracranial atherosclerosis in Asian populations. The degree of BA stenosis in the PPI group was slightly higher than that in the SDPI group, and diabetes mellitus was more prevalent in the PPI group, which further supports the proposal that the main etiology of PPI is probably large artery atherosclerosis. In addition, the prevalence of asymptomatic lacunar infarcts in the pons was higher in the SDPI group, which also supports the role of SVD as the main mechanism underlying SDPI.

In clinical practice, the identification of the etiopathogenesis of stroke might be crucial for guiding appropriate use of early therapies and secondary prevention strategies. For example, if the etiology is large artery atherosclerosis, enhanced antiplatelet and statin treatment is reasonable in the acute phase of stroke, while routine antiplatelet and statins should be given if the etiology involves SVD.

In the present study, we found no significant difference in the prevalence of hypertension between patients with PPI and SDPI, and no increase in the prevalence of hypertension in patients with high total SVD scores. One possible reason for this is that ambulatory blood pressure levels were not evaluated, despite the fact that ambulatory blood pressure is now considered to be an important and modifiable risk factor for SVD [5, 27]. According to the NIHSS scores, neurological deficits in the PPI group were more severe than those in the SDPI group, which is in accordance with the larger maximum diameter of infarcts in the PPI group. This may explain the sample size difference between the PPI and SDPI groups, i.e., due to more severe neurological deficits, more PPI patients were hospitalized in the same period of time than SDPI patients.

Our study suffered from several limitations. 1. There is likely to have been sample selection bias due to the retrospective single-center nature of the investigation. For example, the mean NIHSS score in the sample was low, so patients with severe deficits were not adequately represented in the study. 2. Although the study is the largest retrospective study of an Asian population, the sample was still too small to allow stratification, or to analyze the relationships between isolated pontine infarction, diabetes mellitus, and BA plaques. 3. Embolisms from more proximal arteries, e.g., vertebral arteries and the aortic arc, could not be completely excluded. 4. The definition of BA plaques relevant to the infarcts was based on the normal direction of blood flow in the vessels. Sometimes, especially when there is severe stenosis in the BA, the direction of blood flow can be reversed. In these cases, plaques located in distal segments could be responsible plaques. 5. Contrast-enhanced MRA, which would have mildly affected the precision of the degree of BA stenosis, was not used in the study.

## Conclusions

This study suggests that BA atherosclerosis is probably the major cause of PPI, and SVD may be main mechanism underlying SDPI. This information is likely to be crucial for making appropriate early therapy decisions and implementing appropriate secondary prevention strategies. HR-MRI combined with the total SVD score could play an important role in determining the main pathogenesis in the two types of isolated pontine infarctions, especially in cases with low-grade BA stenosis.

## Abbreviations

BA: Basilar artery
CMBs: Cerebral microbleeds
DWI: Diffusion-weighted imaging
EPVS: Enlarged perivascular spaces
HR-MRI: High-resolution MRI
MRA: MR angiography
NIHSS: The National Institute of Health Stroke Scale
PPI: Paramedian pontine infarct
SDPI: Small deep pontine infarct
SVD: Small vessel disease
SWI: Susceptibility-weighted imaging
WMLs: White matter lesions
